# Systematic review of the characteristics of school-based feasibility cluster randomised trials of interventions for improving the health of pupils in the UK

**DOI:** 10.1186/s40814-022-01098-w

**Published:** 2022-07-02

**Authors:** Kitty Parker, Saskia Eddy, Michael Nunns, ZhiMin Xiao, Tamsin Ford, Sandra Eldridge, Obioha C. Ukoumunne

**Affiliations:** 1 NIHR Applied Research Collaboration South West Peninsula, University of Exeter, Room 2.16, South Cloisters, St Luke’s Campus, 79 Heavitree Rd, Exeter, EX1 2LU UK; 2 Wolfson Institute of Population Health, Barts and The London School of Medicine and Dentistry, Queen Mary University of London, London, UK; 3 College of Medicine and Health, University of Exeter, Exeter, UK; 4 School of Health and Social Care, University of Essex, Colchester, UK; 5 Department of Psychiatry, University of Cambridge, Cambridge, UK; 6 NIHR Applied Research Collaboration South West Peninsula, University of Exeter, Exeter, UK

**Keywords:** Children, Cluster randomised trials, Feasibility study, Pilot study, Public health, Randomised trials, Research methods, Schools, Systematic review, Trial methodology

## Abstract

**Background:**

The last 20 years have seen a marked increase in the use of cluster randomised trials (CRTs) in schools to evaluate interventions for improving pupil health outcomes. Schools have limited resources and participating in full-scale trials can be challenging and costly, given their main purpose is education. Feasibility studies can be used to identify challenges with implementing interventions and delivering trials. This systematic review summarises methodological characteristics and objectives of school-based cluster randomised feasibility studies in the United Kingdom (UK).

**Methods:**

We systematically searched MEDLINE from inception to 31 December 2020. Eligible papers were school-based feasibility CRTs that included health outcomes measured on pupils.

**Results:**

Of 3285 articles identified, 24 were included. School-based feasibility CRTs have been increasingly used in the UK since the first publication in 2008. Five (21%) studies provided justification for the use of the CRT design. Three (13%) studies provided details of a formal sample size calculation, with only one of these allowing for clustering. The median (IQR; range) recruited sample size was 7.5 (4.5 to 9; 2 to 37) schools and 274 (179 to 557; 29 to 1567) pupils. The most common feasibility objectives were to estimate the potential effectiveness of the intervention (*n* = 17; 71%), assess acceptability of the intervention (*n* = 16; 67%), and estimate the recruitment/retention rates (*n* = 15; 63%). Only one study was used to assess whether cluster randomisation was appropriate, and none of the studies that randomised clusters before recruiting pupils assessed the possibility of recruitment bias. Besides potential effectiveness, cost-effectiveness, and the intra-cluster correlation coefficient, no studies quantified the precision of the feasibility parameter estimates.

**Conclusions:**

Feasibility CRTs are increasingly used in schools prior to definitive trials of interventions for improving health in pupils. The average sample size of studies included in this review would be large enough to estimate pupil-level feasibility parameters (e.g., percentage followed up) with reasonable precision. The review highlights the need for clearer sample size justification and better reporting of the precision with which feasibility parameters are estimated. Better use could be made of feasibility CRTs to assess challenges that are specific to the cluster design.

**Trial registration:**

PROSPERO: CRD42020218993.

**Supplementary Information:**

The online version contains supplementary material available at 10.1186/s40814-022-01098-w.

## Background

Cluster randomised trials (CRTs) are studies in which clusters (groups) of individuals are allocated to trial arms, and outcomes are measured on the individual participants [1]. These clusters might be geographical locations (e.g., cities), organisations (e.g., workplaces) or social units (e.g., households). Clusters may be chosen as the randomisation unit for different reasons, including logistical reasons, to prevent contamination that could otherwise occur between trial arms if individuals were randomised, or because the intervention is designed to be administered at the cluster level [2]. CRTs are often used to investigate complex interventions. They usually require more participants and can be more complicated to design, conduct and analyse than individually randomised controlled trials (RCTs) [1–6]. Therefore, it is important to assess the feasibility of the study processes and design uncertainties before a definitive CRT of intervention effectiveness is conducted.

Prior to a definitive trial, a feasibility study can be used to determine whether the research is something that can be done, whether it should be done and how it should be done [7]. Feasibility studies focus on areas of uncertainty in trial delivery, such as the randomisation process, recruitment and follow-up rates, acceptability to the participants of the trial processes and the intervention itself, implementation of the intervention, data collection processes, selection of outcome measures, potential harms related to the intervention and trial, knowledge of parameters that will inform the sample size calculation for the definitive trial, and potential effectiveness of the intervention. The randomised pilot trial is a type of feasibility study that involves conducting the future definitive trial or part of it on a smaller scale [7]. For ease of understanding, this paper refers to randomised pilot trials as feasibility studies. Other types of feasibility study include non-randomised parallel group and single-arm trials, which also focus on developing trial methodology and interventions, and testing processes prior to a full-scale RCT [7, 8]. However, such designs cannot be used to test specific uncertainties such as the randomisation process and the willingness of participants to be randomised. Feasibility CRTs differ from those done in advance of individually RCTs in that they may be used to address concerns that are specific to CRTs, including evaluating the possibility for recruitment bias in studies where clusters are randomised before individual participants are recruited [9] and obtaining estimates of the intra-cluster correlation coefficient (ICC) of the primary outcome to support the calculation of the sample size for the definitive trial, although some authors caution that the resulting estimates will often be imprecise due to the small number of clusters typically included in such studies [10]. Other general feasibility considerations apply at both the cluster and individual levels, such as ease of recruitment, rate of loss to follow-up and acceptability of the intervention. Methodological considerations that are unique to the conduct of feasibility CRTs include the need to take account for clustering when calculating the sample size for and reporting the precision of feasibility parameter estimates from such studies [10].

In recent years, CRTs have been increasingly used to evaluate interventions for improving educational outcomes in schools [11] and complex interventions for improving child health outcomes [12–14]. Schools provide a natural environment in which to recruit and deliver public health interventions to children due to the amount of time they spend there [13]. The CRT design is suited to the natural clustered structure found in schools (pupils within classes within schools), but there are challenges to delivering trials in this setting. For example, schools and teachers often have stretched and limited resources, and implementing an intervention and participating in a trial can be challenging, given that the primary focus of schools is the education of pupils. A recent systematic review of definitive school-based CRTs found that 52% of the studies required a member of school staff to deliver components of the intervention [14]. Obtaining a representative sample of schools is important for external validity and inclusiveness [13], but recruitment of schools and pupils is also a challenge. Another potential feasibility issue regards which type of cluster to randomise in the school setting for a given trial, such as entire schools, year groups, classrooms or teachers. For example, there may be a choice between randomising schools and randomising classrooms; the former would be better to minimise the chance of contamination between trial arms but the latter would have the advantage of a smaller design effect [1] and, therefore, greater power for a fixed total number of recruited pupils [15]. In comparison to the primary care setting, CRTs for evaluating health interventions have only relatively recently been used in schools in the UK and, therefore, there is a smaller pool of experience available from previous studies [1, 14]. Given these uncertainties, feasibility trials have an important role to play in the design and execution of definitive school-based CRTs.

Authors have previously discussed the growing literature described as ‘feasibility’ or ‘pilot’ studies, and the associated methodological challenges [7]. The characteristics of feasibility studies generally [10, 16, 17] and cluster randomised feasibility studies specifically [18, 19] have been summarised, but, to date, no systematic review has focussed on the characteristics of school-based feasibility CRTs for improving pupil health outcomes. The aim of this systematic review is to summarise the key design features and report the feasibility-related objectives of school-based feasibility CRTs in the United Kingdom (UK) that measure health outcomes on pupils. It follows our previous systematic review of full-scale definitive CRTs in the school setting [14]. Through summarising the design features of these studies, the findings of this review will highlight particular areas where improvements could be made to the conduct of feasibility CRTs. The reporting of their feasibility objectives will help identify areas in which better use of such studies could be made to address uncertainties that are specific to the CRT design.

## Methods

### Data sources and search methods

This review has been reported in accordance with the PRISMA (Preferred Reporting Items for Systematic Reviews and Meta-Analyses) statement [20] as evidenced in the PRISMA checklist (see Additional file 1: Table S1) and was registered with PROSPERO (ID CRD: 42,020,218,993; www.crd.york.ac.uk/prospero).

Peer-reviewed school-based feasibility CRTs, indexed on MEDLINE (through Ovid), were the source of data for the review. MEDLINE was systematically searched from inception to 31 December 2020. A pragmatic decision was made to search MEDLINE only due to time constraints and available resources. The search strategy (Table 1) was developed using terms from the MEDLINE search strategy by Taljaard et al. [21] to identify CRTs, and this was combined with *school* concept terms, including the ‘Schools’ MeSH term. This was the same search strategy used in our previous systematic review to identify definitive school-based CRTs [14]. The search was limited to English language papers.

**Table 1:** Systematic review search strategy

Search strategy
**Terms for Randomised Controlled trials:**
1. random:.mp
2. trial.ab, kw, ti
**Cluster design-related terms:**
3. “cluster*”.ab, kw, ti
4. “communit*”.ab, kw, ti
5. group*adj2 random*.ab, kw, ti
6. 3 OR 4 OR 5
**School terms:**
7. exp Schools/
8. School*.ab, kw, ti
9. 7 OR 8
**Final search stages:**
10. 1 AND 2 AND 6 AND 9
11. 10 limited to English language

### Inclusion and exclusion criteria

The review included school-based feasibility CRTs that measured health outcomes on pupils and were conducted in the UK. It focussed on the UK to align with available resources and to summarise data from a single education system relevant to the research team. The population of included studies was pupils attending pre-school, primary or secondary school in the UK. ‘Pre-school’ was defined as an organisation offering early childhood education to children before they begin compulsory education (i.e., primary school). This included nursery schools and kindergartens. Eligible clusters could be any school-related unit (e.g., schools, classes, year groups). Studies that randomised school-related units as well as other types of clusters (e.g., towns, hospitals, households) were eligible for inclusion in the review as long as the results of the study were shown separately for the school clusters (i.e., the authors did not pool results across the different types of clusters). Any health-related intervention(s) were eligible. The primary outcome had to be measured on pupils and be health related. Studies with education-related primary outcomes were excluded. All types of CRT design were eligible, including parallel group, factorial, crossover and stepped wedge trials.

Only randomised external feasibility studies were included in the systematic review. The definition of feasibility study used to identify eligible papers was that used by Eldridge et al. [7] which states “A feasibility study asks whether something can be done, should we proceed with it, and if so, how.” Therefore, eligible studies had to be assessing some element of feasibility in the intervention and/or trial methodology, ahead of a definitive trial. This was determined by looking for the terms, ‘pilot’, ‘feasibility’ or ‘explanatory’ in the title and abstract and by examining the aims and objectives of each study. Internal pilot studies that are part of the actual definitive trial, where the data from the pilot phase are included in the main analysis [22] were excluded. Non-randomised parallel group feasibility studies and single-arm feasibility studies were excluded. Definitive CRTs were not eligible for inclusion in this review.

If there was more than one publication of the results for an eligible feasibility CRT, the paper presenting quantitative results related to the feasibility objectives was designated the key study report (index paper) and used for data extraction. Papers that did not report the results of the feasibility objectives were excluded along with protocol/design articles, cost-effectiveness/economic evaluations and process evaluations.

### Sifting and validation

Titles and abstracts were downloaded into Endnote [23] and screened by two independent reviewers (KP & SEd/OU) for eligibility against inclusion criteria. Studies for which inclusion status was uncertain were included for full-text screening. Full-text articles were assessed against inclusion criteria by two reviewers (KP & SEd) using a pre-piloted coding method. Any uncertainties were resolved by consulting a third reviewer (OU).

### Data extraction

The data extraction form was pre-piloted in Microsoft Excel by KP and SEd. One investigator (KP) extracted data from all included studies. A second reviewer (SEd or OU) independently extracted data for validation. If there was uncertainty regarding a particular article, the data obtained were checked by another member of the team (MN) and resolved by further discussion.

The items of information extracted are listed as follows:

- *Publication details*: year of publication, journal name, funding source and trial registration status.

- *Setting characteristics*: country (England, Scotland, Wales, Northern Ireland) in which the trial took place, school level, types of school recruited and participant information.

- *Intervention information*: health area, intervention description and type of control arm.

- *Primary outcome information*: name of primary outcome.

- *Study design*: justification for using cluster trial design, type of cluster, method of randomisation, timing of randomisation of clusters relative to recruitment of pupils, number of trial arms, allocation ratio and length of follow-up.

- *Sample size information*: justification for sample size, targeted number of schools, clusters and pupils; number of recruited schools, clusters and pupils.

- *Objectives of feasibility study*: test randomisation process (yes/no), test willingness to be randomised (at cluster and/or individual levels) (yes/no), estimate recruitment rate (at cluster and/or individual levels) (yes/no), estimate retention/follow-up rate (at cluster and/or individual levels) (yes/no), test implementation of the intervention (yes/no), test compliance with the intervention (yes/no), assess acceptability of the intervention (at cluster and/or individual levels) (yes/no), assess acceptability of trial procedures (at cluster and/or individual levels) (yes/no), test the feasibility of blinding procedures (yes/no), test data collection process (yes/no), test outcome measures (yes/no), estimate standard deviation for continuous outcomes (or control arm rate for binary outcomes) (yes/no), test consent procedures (yes/no), identify potential harms (yes/no), estimate potential effectiveness of intervention (yes/no), estimate costs of delivering the intervention (yes/no), estimate the intra-cluster correlation coefficient (ICC) of the primary outcome (yes/no) and calculate the sample size required for the definitive trial (yes/no). Only formal feasibility objectives were extracted; these were obtained from the Background and Methods sections of the included articles.

- *Ethics and consent procedures*: Was ethical approval provided? (yes/no).

- *Other design characteristics of methodological interest: *analysis method used to estimate potential effectiveness of the intervention, baseline cluster-level characteristics, ICC estimates (and 95% confidence intervals (CIs)) and whether study concluded that a definitive trial is feasible (yes/yes (with modifications)/no).

### Data analysis

Study characteristics were described using medians, interquartile ranges (IQRs) and ranges for continuous variables, and numbers and percentages for categorical variables, using Stata 17 software [24]. Formal quality assessment of the papers was not performed as it was not necessary for summarising characteristics of studies. However, some of the data extracted and summarised in the review are indicative of the reporting quality of included studies based on the items in the CONSORT extension for both CRTs [25] and pilot studies [26]. This includes details on the rationale for using the CRT design, the rationale for the target sample size and ethical approval procedures.

## Results

### Search results

**Fig. 1:**
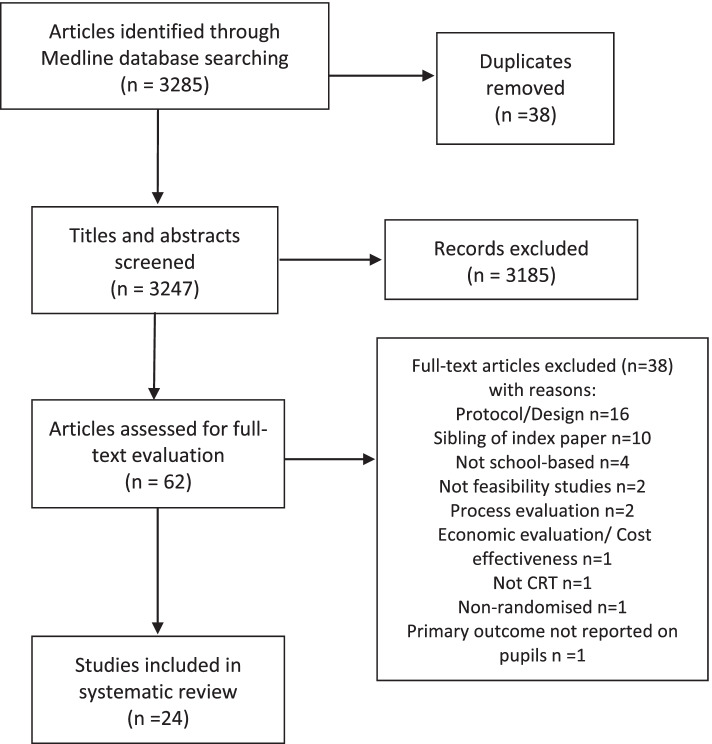
PRISMA flowchart summarising the results of the literature search and screening for eligibility

After deduplication, 3247 articles were identified through MEDLINE, 62 were eligible for full-text screening and 24 were included in the review [27–50]. Out of 38 excluded studies, 28 did not meet the inclusion criteria, and 10 met inclusion criteria but were excluded as they described the same study as a designated ‘index paper’. The PRISMA flow diagram [20] is shown in Fig. 1.

### Study characteristics

**Fig. 2:**
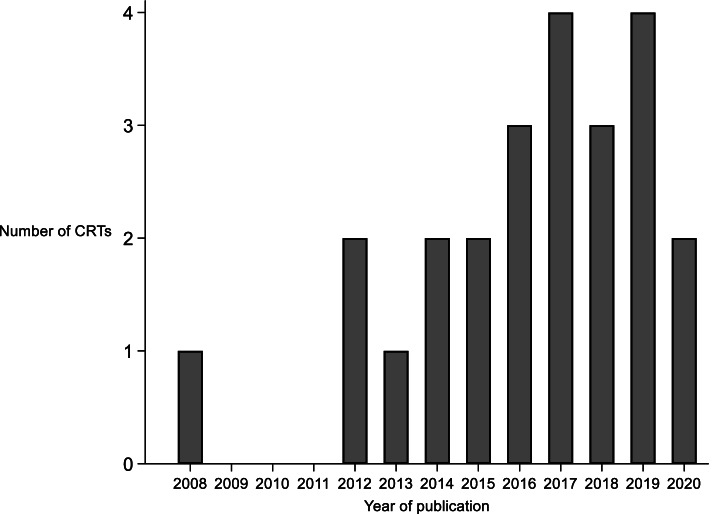
Published feasibility CRTs indexed on MEDLINE from inception to 31st December 2020 (*N* = 24)

School-based feasibility CRTs for health interventions on pupils have been increasingly used in the UK since the first publication in 2008 (Fig. 2). Included articles were published across 11 different journals: *Pilot and Feasibility Studies* (*n* = 5), *International Journal of Behavioural Nutrition and Physical Activity* (*n* = 4), *Public Health Research* (*n* = 4), *BMJ Open* (*n* = 3), *Health Technology Assessment* (*n* = 2), *Archives of Disease in Childhood* (*n* = 1), *BMC Public Health* (*n* = 1), *British Journal of Cancer* (*n* = 1), *British Journal of Psychiatry* (*n* = 1), *Prevention Science* (*n* = 1) and *Trials* (*n* = 1). Ten articles described their study as a ‘pilot trial’, six as a ‘feasibility trial’, four as a ‘feasibility study’, two as an ‘exploratory trial’, one as a ‘pilot feasibility trial’ and one as a ‘pilot study’. Twelve (50%) studies were funded by the *National Institute for Health Research*. Eight (33%) studies were registered prospectively, thirteen (54%) retrospectively, and three (13%) did not state registration status.

Tables 2 and 3 summarise the characteristics of included studies.

**Table 2:** Characteristics of included studies (*N* = 24)

Author	Year of publication	School level	Cluster unit	Health area
Kipping [39]	2008	Primary	Schools	Physical activity and nutrition
Jago [36]	2012	Secondary	Schools	Physical activity
Lloyd [40]	2012	Primary	Schools	Physical activity and nutrition
Sharpe [48]	2013	Secondary	Classes	Body image
Jago [37]	2014	Primary	Schools	Physical activity
Newbury-Birch [44]	2014	Secondary	Schools	Alcohol misuse
Bonell [28]	2015	Secondary	Schools	Bullying
Segrott [47]	2015	Primary	Schools	Alcohol misuse
Barber [27]	2016	Pre-school	Schools	Physical activity
Corder [31]	2016	Secondary	Schools	Physical activity
Wright [50]	2016	Primary and secondary	Schools	Behavioural/social difficulties (Autism)
Forster [33]	2017	Secondary	Schools	Sexual health (Cancer)
Ginja [35]	2017	Primary	Schools	Physical activity
McSweeney [42]	2017	Pre-school	Schools	Physical activity and nutrition
White [49]	2017	Secondary	Schools	Illicit drug misuse
Carlin [29]	2018	Secondary	Schools	Physical activity
Lohan [41]	2018	Secondary	Schools	Sexual health
Sebire [46]	2018	Secondary	Schools	Physical activity
Corepal [32]	2019	Secondary	Schools	Physical activity
Gammon [34]	2019	Secondary	Schools	Physical activity
Johnstone [38]	2019	Primary	Schools	Physical activity
Sahota [45]	2019	Primary	Schools	Physical activity and nutrition
Clemes [30]	2020	Primary	Schools	Physical activity
Meiksin [43]	2020	Secondary	Schools	Dating and relationship violence

**Table 3:** Summary of methodological characteristics of included studies (*N* = 24)

**Characteristic**	N	**Statistic**
***Setting***		
*Country*	24	
England, n (%)		18 (75)
Scotland, n (%)		1 (4)
Wales, n (%)		2 (8)
Northern Ireland, n (%)		3 (13)
*School types that were included *[51]*[Accessed 1*^*st*^* September 2021] *^a^	15	
State, n (%)		14 (93)
Academy, n (%)		3 (20)
Voluntary aided, n (%)		1 (7)
Foundation, n (%)		1 (7)
Faith, n (%)		1 (7)
Grammar, n (%)		1 (7)
Independent, n (%)		1 (7)
***Intervention***		
*Type of intervention *[1] ^b^	24	
Individual-cluster, n (%)		2 (8)
Professional-cluster, n (%)		18 (75)
External-cluster, n (%)		8 (33)
Cluster–cluster, n (%)		23 (96)
Multifaceted, n (%)		21 (88)
*Intervention components*^c^	24	
Resources and materials for schools, n (%)		11 (46)
Classroom lessons, n (%)		10 (42)
Physical activity lessons, n (%)		5 (21)
Incentive scheme, n (%)		4 (17)
Change in school/classroom environment, n (%)		4 (17)
Peer support, n (%)		3 (13)
Support for parents/guardians, n (%)		3 (13)
Goal setting, n (%)		2 (8)
Staff training, n (%)		2 (8)
Home activities, n (%)		2 (8)
Extracurricular physical activity, n (%)		2 (8)
Parent’s evenings, n (%)		1 (4)
Drama workshops, n (%)		1 (4)
Funding, n (%)		1 (4)
School action group formation, n (%)		1 (4)
School club sessions, n (%)		1 (4)
Screening, n (%)		1 (4)
Feedback, n (%)		1 (4)
Motivational interviews, n (%)		1 (4)
Interactive sessions, n (%)		1 (4)
Discussions with parents/guardians, n (%)		1 (4)
Gamification (competitive) techniques, n (%)		1 (4)
*Type of control group*	24	
Usual care, n (%)		21 (88)
Active, n (%)		2 (8)
Two control groups (one usual care and one active control), n (%)		1 (4)
***Study design***		
*Justification for CRT design*	24	
Yes, n (%)		5 (21)
*Type of randomisation*	24	
Completely randomised, n (%)		11 (46)
Minimisation, n (%)		5 (21)
Stratified, n (%)		4 (17)
Matched pair, n (%)		3 (13)
Constrained [52, 53], n (%)		1 (4)
*Number of trial conditions*	24	
Two, n (%)		21 (88)
Three, n (%)		2 (8)
Four, n (%)		1 (4)
*Length of follow-up*	24	
Up to 6 months, n (%)		11 (46)
7 to 12 months, n (%)		8 (33)
13 to 18 months, n (%)		3 (13)
More than 18 months, n (%)		1 (4)
Not stated, n (%)		1 (4)
*Were pupils recruited before randomisation of clusters?*	24	
Pupils recruited before randomisation, n (%)		12 (50)
Pupils recruited after randomisation, n (%)		4 (17)
Unclear, n (%)		8 (33)
*Were baseline cluster-level characteristics reported?*	24	
Yes, n (%)		13 (54)
***Ethical approval***		
*Was ethical approval obtained?*	24	
Yes, n (%)		22 (92)
No, n (%)		1 (4)
Not stated, n (%)		1 (4)
***Sample size***		
*Type of justification for sample size*	24	
Formal sample size calculation^d^, n (%)		3 (13)
Other justification, n (%)		19 (79)
Not stated, n (%)		2 (8)
*Target number of schools, median (IQR; range)*	18	7.5 (5 to 8; 2 to 20)
*Target number of clusters, median (IQR; range)*	18	7.5 (5 to 8; 2 to 20)
*Target number of pupils, median (IQR; range)*	13	320 (150 to 1200; 50 to 1852)
*Achieved number of schools, median (IQR; range)*	24	7.5 (4.5 to 9; 2 to 37)
*Achieved number of clusters, median (IQR; range)*	24	8 (5.5 to 9.5; 2 to 37)
*Achieved number of pupils, median (IQR; range)*	24	274 (179 to 557; 29 to 1567)
*Achieved mean cluster size, median (IQR; range)*	24	35.9 (24 to 89.4; 1.4 to 237.7)
***Objectives of the feasibility study***		
*Feasibility objectives*	24	
Test randomisation process, n (%)		3 (13)
Test data collection process, n (%)		8 (33)
Test willingness to be randomised (at cluster level and/or individual levels), n (%)		4 (17)
Estimate recruitment percentage (at cluster level and/or individual levels), n (%)		15 (63)
Estimate follow-up percentage (at cluster level and/or individual levels), n (%)		15 (63)
Test implementation of intervention, n (%)		10 (42)
Test compliance with intervention, n (%)		6 (25)
Assess acceptability of intervention (at cluster level and/or individual levels), n (%)		16 (67)
Assess acceptability of trial procedures (at cluster level and/or individual levels), n (%)		6 (25)
Test the feasibility of blinding procedures, n (%)		0 (0)
Test outcome measures, n (%)		14 (58)
Estimate standard deviation of continuous outcomes or control arm rate for binary outcomes, n (%)		1 (4)
Test consent procedures, n (%)		0 (0)
Identify potential harms, n (%)		3 (13)
Assess potential effectiveness of intervention, n (%)		17 (71)
Estimate intervention cost, n (%)		7 (29)
Estimate the ICC of the primary outcome, n (%)		2 (8)
Estimate sample size for definitive trial, n (%)		5 (21)
***Other study characteristics of methodological interest***		
*Analysis method for estimating potential effectiveness*	24	
Individual-level analysis that allows for clustering, n (%)		9 (38)
Cluster-level analysis, n (%)		4 (17)
Did not account for clustering, n (%)		4 (17)
Not stated, n (%)		3 (13)
Did not estimate potential effectiveness, n (%)		4 (17)
*P-value reported for effectiveness*	24	
Yes, n (%)		8 (33)

^a^Some studies included more than one school type. This is the number of studies that included specific types of school. State schools receive funding through their local authority or directly from the government. The most common ones are local authority, foundation and voluntary aided school which are all funded by the local authority. Academies are run by government and not-for-profit trusts, and are independent of local authority. Grammar schools are run by local authorities but intake is based on assessment of the pupils’ academic ability. Special schools cater for pupils with special educational needs. Faith schools follow the national curriculum but can decide what they teach in religious studies. Independent schools follow the national curriculum but charge fees for attending pupils

^b^Intervention type has been described using the typology of Eldridge and Kerry [1]. ‘Individual-cluster’ interventions contain components that are aimed at the individual level (e.g., goal setting). ‘Professional-cluster’ interventions contain components that are delivered by a professional or person internal to the cluster (e.g., teacher, pupils). ‘External-cluster’ interventions contain components that require people external to the cluster to deliver the intervention (e.g., research staff, community support consultant). ‘Cluster–cluster’ interventions contain components that have to be delivered at the cluster level (e.g., classroom lessons). ‘Multifaceted’ interventions contain components across more than one of the ‘individual-cluster’, ‘professional-cluster’, ‘external-cluster’ and ‘cluster–cluster’ categories

^c^Examples of each intervention component are provided for ease of understanding. Resources and materials (e.g., a resource box comprising food models, food mats, food cards, DVDs, and books); Classroom lessons (e.g., interactive film-based sexual-health lesson); Physical activity lessons (e.g., active play sessions, brisk walking programme during the school day); Incentive schemes (e.g., lottery-based incentive scheme to promote active travel to school); Peer support (e.g., informal peer-led smoking prevention); Change in school/classroom environment (e.g., sit-stand desks to replace standard desks, challenging attitudes and perceived norms concerning gender stereotypes and dating and relationship violence); Support for parents/guardians (e.g., information sheets about health eating habits); Goal setting (e.g., goal setting to engage and support schools); Staff training (e.g., staff training in restorative school action group formation); Home activities (e.g., home activities that encourage pupils to be more active, eat more nutritious foods, and spend less time in screen-based activities); Extracurricular physical activity (e.g., staff delivered after-school physical activity programme); Drama workshops (e.g., interactive drama workshops); School action group formation (e.g., to address bullying and aggression within schools); School club sessions (e.g., health eating club); Screening (e.g., alcohol screening and brief intervention to reduce hazardous drinking in younger adolescents); Feedback (e.g., feedback about pupil’s drinking habits); Motivational interviews (e.g., motivational interviewing techniques to prevent alcohol misuse); Interactive sessions (e.g., interactive sessions with school learning mentors to prevent alcohol misuse); Discussions with parents/guardians (e.g., guided discussions conducted with parents); Gamification (competitive) techniques (e.g., gamification techniques to promote physical activity)

^d^In one study, the sample size was based on being able to estimate feasibility parameters with a pre-specified level of precision. Two studies based their sample size on a definitive test of intervention effectiveness

#### Setting

Three quarters of studies (*n* = 18; 75%) took place in England. Just over half (*n* = 13; 54%) took place exclusively in secondary schools, 8 (33%) took place exclusively in primary schools, 2 (8%) exclusively in pre-schools and 1 (4%) study included both primary and secondary schools. Fifteen (63%) studies provided information about the types of schools included in their sample and, of these, 14 (93%) included “state” schools.

#### Intervention and control type

Eleven (46%) studies delivered interventions for improving physical activity, 4 (17%) in physical activity and nutrition, 2 (8%) in alcohol misuse, 2 (8%) in sexual health and 1 (4%) in each of illicit drug misuse, bullying, behavioural/social difficulties, body image, and dating and relationship violence.

The main types of intervention components included resources and materials for schools (*n* = 11; 46%), classroom lessons (*n* = 10; 42%) and physical activity lessons (*n* = 5; 21%). Almost all studies (*n* = 23, 96%) had intervention components that had to be delivered to entire clusters (‘cluster–cluster’ interventions [1] (pages 25 to 30))—e.g., classroom-delivered lessons [48] and physical activity sessions [27]. Two (8%) had intervention components that were directed at individual pupils (‘individual-cluster’ interventions [1])—e.g., goal-setting [40, 50]. Eighteen (75%) had intervention components that were delivered by a professional or person internal to the cluster (‘professional-cluster’ interventions [1])—e.g., teachers [34], member of school staff [27] and fellow pupils/peers [46]. Eight studies (33%) had intervention components that were delivered by someone external to the cluster (‘external-cluster’ interventions [1])—e.g., ‘active play practitioners’ [38], researchers [41] and dance teachers [36].

The most common type of control arm was usual care (*n* = 21; 88%). Two (8%) studies used an active control arm, and one (4%) study had two control arms (a usual care arm and an active control arm).

#### Study design

Justification for the use of the CRT design was provided in only 5 (21%) studies. The reasons given were that the intervention was designed to be delivered to entire clusters [30, 47, 48] and to minimise contamination between trial arms [44, 50]. Twenty-three (96%) studies randomised schools and the remaining study randomised classrooms [48]. In the latter study [48], random allocation was carried out at the level of the classroom for ‘pragmatic considerations’. Thirteen (54%) studies used some form of restricted allocation to balance cluster characteristics between the trial arms.

Most studies (*n* = 21; 88%) had two trial arms and most allocated clusters in a 1:1 ratio (*n* = 17; 71%). The median (IQR; range) length of follow up was 7 (3 to 12; 2 to 24) months.

Twelve (50%) studies recruited pupils before randomisation of clusters, four (17%) randomised clusters before recruiting pupils, and in eight (33%) studies, it was unclear whether or not randomisation occurred before pupils were recruited. Only 13 (54%) studies reported baseline characteristics of the schools.

#### Ethical approval

Ethical approval was obtained and reported in 22 (92%) studies. One study stated that ethical approval was sought but the local research committee said it was not required as the study did not involve patients or NHS staff. The remaining study did not state whether ethical approval was obtained.

#### Sample size

Of the 24 studies included in this review, three (13%) provided details of a formal sample size calculation. One of these studies based their sample size on being able to estimate feasibility parameters (e.g., participation rates, questionnaire response rates) with a specified level of precision [33], and the other two studies based their sample size on power to detect a specified intervention effect [29, 48]. Only one (4%) study allowed for clustering in their sample size calculation [48]. Nineteen studies provided informal justification for their sample size calculation, based on one or more reasons: seven (29%) studies based their target sample size on recommendations from previous articles, six (25%) studies stated that a formal sample size calculation was not needed, four (17%) studies said their target sample size was determined by resource and/or time constraints, three (13%) studies provided a general statement that their sample size was considered sufficient to address the objectives of the feasibility CRT, and one (4%) study aimed to recruit as many clusters and participants as possible. Two (8%) studies did not provide any justification for their choice of sample size.

The median (IQR; range) target sample size was 7.5 (5 to 8; 2 to 20) schools, 7.5 (5 to 8; 2 to 20) clusters and 320 (150 to 1200; 50 to 1852) pupils. The median (IQR; range) achieved sample size was 7.5 (4.5 to 9; 2 to 37) schools, 8 (5.5 to 9.5; 2 to 37) clusters and 274 (179 to 557; 29 to 1567) pupils. Two studies included just 2 schools, with 1 school allocated to each trial arm [34, 35]. The studies that reported both targeted and achieved recruitment numbers at the cluster (*n* = 18) and pupil (*n* = 13) levels achieved those targets in 94% and 46% of studies, respectively.

#### Objectives of feasibility study

Formal feasibility objectives were specified by all 24 studies (summarised in Table 3). Of the 18 objectives assessed in this review, the median (IQR; range) number addressed per study was 5 (4 to 7.5; 1 to 11). The most common objectives were to estimate the potential effectiveness of the intervention (*n* = 17; 71%; including two studies that sought to undertake a definitive test of effectiveness [29, 48]), assess acceptability of the intervention (*n* = 16; 67%), estimate the recruitment rate (*n* = 15; 63%), estimate the retention/follow-up rate (*n* = 15; 63%) and test outcome measures (*n* = 14; 58%). Two studies included estimation of the intra-cluster correlation coefficient of the primary outcome to be used in the planned definitive study as a formal objective of the feasibility study. No studies tested the feasibility of blinding or consent procedures. All studies reported additional feasibility outcomes beyond those formally stated as objectives.

The following feasibility objectives were stated specifically at the level of the cluster: assess acceptability of the intervention (*n* = 10; 42%), estimate retention/follow-up rate (*n* = 7; 29%), estimate recruitment rate (*n* = 6; 25%), assess willingness to be randomised (*n* = 4; 17%) and assess acceptability of the trial procedures (*n* = 3; 13%). One (4%) feasibility CRT had the formal objective of assessing the appropriateness of cluster randomisation [50]. None of the feasibility studies used their research to assess the type of cluster that should be randomised. Of the 4 studies that randomised clusters before recruiting pupils, none investigated the possibility of recruitment bias.

Analyses were undertaken to investigate if the target sample size differed according to whether or not the studies addressed specific feasibility objectives. Many objectives were only formally stated in a small number of studies; therefore, it was hard to identify clear patterns in the data. The twelve studies that assessed potential effectiveness aimed to recruit a median (IQR; range) of 7 (3.5 to 8; 2 to 20) schools, similar to the targeted recruitment in the remaining studies (7.5 (6 to 8; 5 to 12)).

All studies reported estimates of feasibility parameters, but, other than for potential intervention effectiveness, cost-effectiveness and the intra-cluster correlation coefficient, no studies quantified the precision of these estimates. Five of the eight (63%) studies that reported estimates of the ICC for the provisional primary outcome of the planned definitive study provided 95% confidence intervals (95 CIs) for these. Table 4 reports the ICC estimates. As expected the 95% confidence intervals were generally wide given that the sample size is small for estimating the ICC. Notably, however, the upper bound for two ICC estimates was only 0.03, which provides useful information on plausible true values of the parameter despite those studies having only 6 [46] and 19 [39] clusters.

**Table 4:** Reported intra-cluster correlation coefficients for primary outcomes (*N* = 8)

Author (Year)	Cluster unit	Health area	Outcome	Outcome type	ICC (95% CI)
Jago (2012) [36]	Schools	Physical activity	MVPA (minutes per weekday)	Continuous	0.018 (< 0.001 to 0.087)
Jago (2014) [37]	Schools	Physical activity	MVPA (minutes per weekday)	Continuous	0.0653 (0.00091 to 0.12977)
Kipping (2008) [39]	Schools	Physical activity and nutrition	Minutes spent on screen-based activities	Continuous	0.01 (0 to 0.03)
Lloyd (2012) [40]	Schools	Physical activity and nutrition	BMI SD score	Continuous	0.04 (0 to 0.15)
Sahota (2019) [45]	Schools	Physical activity and nutrition	Healthy nutrition and physical activity knowledge	Continuous	0.07 (Not provided)
Sebire (2018) [46]	Schools	Physical activity	MVPA (minutes per weekday)	Continuous	< 0.0001 (0.0 to 0.03)
Segrott (2015) [47]	Schools	Alcohol misuse	Drinking initiation	Binary	0.112 (Not provided)
White (2017) [49]	Schools	Illicit drug misuse	Lifetime illicit drug use	Binary	0.003 (Not provided)

*BMI* Body mass index, *CI* Confidence interval, *ICC* Intra-cluster correlation coefficient, *MVPA* Moderate to vigorous physical activity, *SD* Standard deviation

Of the 20 studies that reported intervention effect estimates, nine (45%) used an adjusted individual-level analysis method to allow for clustering, 4 (20%) used a cluster-level analysis method, four (20%) did not allow for clustering and three (15%) did not state the analytical method. Eight studies reported *p* values with the intervention effect estimate, contrary to published guidance for feasibility studies [25, 26].

Eleven (46%) studies concluded that the definitive trial was feasible, 11 (46%) said the definitive trial would be feasible with modifications and two (8%) said that the planned study was not feasible. Through searching the literature and personal correspondence with the authors, it was established that of the 24 feasibility CRTs included in the review, 11 are known to have progressed to definitive trials [28, 29, 31, 36, 39–41, 44, 46, 49, 50]. Of these, nine had concluded that the definitive trial was feasible, and two had concluded that the definitive trial would be feasible with modifications.

## Discussion

### Main findings

This is the first systematic review to summarise the characteristics and objectives of school-based feasibility CRTs of interventions to improve pupil health outcomes in the UK. The review found an increase in such studies since the earliest included paper was published in 2008. This mirrors the increase in definitive CRTs in this area reported in our parallel review [14] and highlights the rising popularity of health-based CRTs in the school-setting. The increase in feasibility CRTs may partly be due to the publication of the 2006 MRC guidelines for the evaluation of complex interventions [54] which highlights the importance of conducting feasibility studies ahead of full-scale trials. The relatively large number of feasibility CRTs with interventions for increasing physical activity indicates the growing importance of adolescent physical activity as a public health priority, and the use of schools in order to deliver these types of intervention [55]. The review of school-based definitive CRTs also reflected the increasing use of the design to evaluate physical activity interventions [14]. Based on what was observed in the review of definitive school-based CRTs, there were fewer than expected feasibility studies in the area of socioemotional functioning. This is despite the increased awareness of the prevalence of these health conditions and research funding in this area [56].

A previous review of feasibility CRTs found that, among other objectives, assessing the implementation of the intervention (*n* = 9, 50%) was the most common [18]. The studies included in the current review sought to address a range of feasibility objectives; most commonly estimating potential effectiveness of the intervention, assessing acceptability of the intervention, estimating the recruitment and follow-up rates and testing the outcome measures. It was notable, however, that few studies formally stated objectives that were related to uncertainties that are unique to the cluster design. This finding is similar to another review of feasibility CRTs which also stated that few studies investigated issues specific to the complexities of the design [19]. None of the 4 studies that randomised clusters before recruiting pupils investigated the potential for recruitment bias as a feasibility objective. In the current review, only one study assessed whether a cluster design was needed, and none used the research to decide on the type of school-based cluster (e.g., school versus classroom) that was best to randomise. It may be the case that the need for cluster randomisation and the appropriate type of cluster to allocate had a strong theoretical basis, negating the need for empirical justification, but only 5 of the 24 studies provided a rationale for the cluster design even though the CONSORT extension for CRTs [25] recommends reporting this.

The studies included in this review were heterogeneous in their formal feasibility objectives, and this may have influenced specific features of their design, such as sample size and length of follow-up. The designs may also have been influenced by other factors such as budget, time and practical constraints.

Only three (13%) studies in the review reported details of a formal calculation for the target sample size [29, 33, 48], and only one accounted for clustering in the sample size calculation [48]. These results are similar to that found in a previous systematic review of feasibility CRTs which reported that only one of the 18 studies reported a formal sample size calculation based on the primary feasibility objective [18]. A quarter of the included papers in the current review stated that a formal sample size calculation was not needed, and some authors have argued that it is not always appropriate in feasibility studies [16]. In a recent review of current practice in feasibility studies, only 36% reported sample size calculations [57]. Also, when surveyed, some journal editors stated they were willing to accept pilot studies for publication that did not report a sample size calculation [57]. The precision with which parameters are estimated in feasibility CRTs should be reported, especially given the small number of clusters that are typically included in such studies. Despite this, apart from when assessing the effectiveness of the intervention, cost-effectiveness and estimating the ICC, this was not done by any papers in the current review. Correspondingly, a formal sample size calculation based on the feasibility objectives that allows for clustering [10] is appropriate to estimate parameters precisely and, therefore, minimise the uncertainty regarding the assumptions that are made for the subsequent definitive study [16, 57].

Our review found the median number of clusters recruited (eight) was similar to a previous review of feasibility studies [18]. Based on results from a simulation study, it has been suggested that as many as 30 or more clusters may be required in a feasibility CRT in order to avoid downwardly biased and imprecise estimates of the number of clusters required to test the intervention effect in the subsequent definitive CRT; this is largely due to the imprecision with the ICC is estimated in the feasibility study [10]. The current review found only one study that recruited more than 30 clusters [50], and it is difficult to achieve this level of recruitment due to funding and practical constraints. Smaller feasibility studies may, however, still provide informative estimates of many parameters. Two of the feasibility studies in the review, despite including only 6 [46] and 19 [39] clusters, were able to estimate the intra-cluster correlation coefficient with a 95% confidence interval upper bound of 0.03, which could rule out the need for unattainably large sample sizes in the definitive study. Many studies report feasibility objectives in the form of percentages (e.g., follow-up rates, intervention adherence rates). Eldridge and colleagues [10] provide formulae for calculating the sample size required in feasibility CRTs to estimate percentages based on individual-level characteristics (e.g., whether the pupil was followed up) with a confidence interval of specified width, whilst allowing for clustering. Assuming the ICC for the feasibility characteristic is 0.05, a study with 8 schools and 240 pupils (an average sample size based on the findings in the current review) is large enough to estimate the percentage with a margin of error no greater than 10 percentage points based on a 95% confidence interval. There will generally be little precision for estimating percentages based on cluster-level characteristics since this is determined by the, typically, small number of schools (clusters) in feasibility studies.

Another important reason to recruit sufficient clusters to feasibility CRTs is to assess how the intervention might be implemented and the trial delivered in a range of different types of cluster [18]. Parameter estimates will only be useful to the extent that the clusters and individuals in the feasibility study are broadly representative and reflect the diversity of the population from which the sample in the definitive trial will be drawn [18]. In the context of school-based trials, important aspects of representativeness include single sex versus co-educational schools, state versus independent schools, and deprived versus non-deprived areas. In the current review, only 54% of studies reported baseline characteristics of the schools, although this is higher than found in a previous systematic review of feasibility CRTs where only 11% of studies reported baseline cluster-level characteristics [18].

The current systematic review found that of the 13 studies that reported both targeted and achieved numbers of pupils recruited, those targets were only achieved in 46% of studies. Our previous systematic review of definitive school-based CRTs found that only 77% of studies achieved their target recruitment of pupils [14]. The facilitators and barriers to the recruitment and retention of pupils to school-based CRTs have been discussed in detail in the literature [58–60], including the type of intervention being offered and the perceived benefits of the study (e.g., sexual education) [58, 60], lack of time [58], incompatibility of the intervention with the needs of pupils or parents or with the school’s ethos [58] and a lack of incentivisation [59].

### Strengths and limitations

A strength of the review is that a predefined search strategy was used to identify feasibility cluster randomised trials in the school setting. The protocol was publicly available prior to conducting the review. Screening, piloting of the data extraction form and data extraction were conducted by two independent reviewers. A pragmatic decision was made to limit the review to the UK in order to align with available resources and to make it more focused.

A limitation is the decision to use only the MEDLINE database. MEDLINE was chosen as health-based studies were the focus of this review. We acknowledge that further articles may have been found by searching other databases, grey literature and through citation searching. The search strategy was translated in EMBASE, DARE, PsycINFO and ERIC databases to search for additional eligible school-based CRTs published between 2017 and 2020 and resulted in identification of only one further unique eligible article. Therefore, we feel the pragmatic approach to only use MEDLINE to perform this search did not result in omission of a significant body of relevant evidence.

The systematic review only included feasibility studies that used the cluster randomised trial design and not other types, such as non-randomised parallel group and single-arm feasibility studies. We focussed on CRTs because we were interested in studies that could be used to assess a wide range of uncertainties for definitive CRTs, but we acknowledge that the systematic review may, therefore, not include some relevant knowledge of practice in non-randomised feasibility studies. While the approach used was not comprehensive, it enabled us to efficiently identify studies of interest that were undertaken in advance of planned definitive CRTs.

A further limitation of the review is that data were not extracted on consent procedures used by the included studies. As found in our previous review of definitive school-based CRTs [14], this information was inconsistently reported across studies making it challenging to summarise. This highlights the need for more comprehensive reporting of the consent procedures in these studies.

## Conclusions

Cluster randomised feasibility studies are increasingly used in the school setting to test feasibility prior to definitive trials. Although these studies usually include few schools, the average sample size of those included in this review would be large enough to estimate percentages based on pupil characteristics that are used to address feasibility objectives (e.g., the percentage followed up) with a reasonable level of precision. The review has highlighted the need for clearer justification for the target sample size of school-based feasibility CRTs and to report the precision with which feasibility parameters are estimated in these studies. The characteristics of the recruited schools in feasibility CRTs could be better described to help understand the extent to which the feasibility parameter estimates are applicable to the planned definitive trial and other future similar trials. Finally, better use could be made of feasibility CRTs in the area of school-based pupil health research to assess challenges that are specific to the cluster trial design.

## Supplementary Information

**Additional file 1: Table S1. **PRISMA checklist.

## Data Availability

The datasets generated and/or analysed during the current study are not publicly available because they are also being used for a wider ongoing programme of research but are available from the corresponding author on reasonable request.

## Abbreviations

BMI: Body mass index
CI: Confidence interval
CRT: Cluster randomised trial
DARE: Database of Abstracts of Reviews of Effects
EMBASE: Excerpta Medica Database
ERIC: Education Resources Information Center
ICC: Intra-cluster correlation coefficient
IQR: Interquartile range
MEDLINE: Medical Literature Analysis and Retrieval System Online
MeSH: Medical Subject Headings
MVPA: Moderate to vigorous physical activity
PRISMA: Preferred Reporting Items for Systematic Reviews and Meta-Analyses
PsycINFO: Psychological Information Database
SD: Standard deviation
UK: United Kingdom
